# Induction of Carotenoid Accumulation by Salt Stress in a Newly Isolated *Dysmorphococcus* Strain from Antalya, Türkiye

**DOI:** 10.3390/ijms27219416

**Published:** 2026-10-22

**Authors:** Waed Mohammad Alnsour, Xenie Johnson, Münevver Aksoy

**Affiliations:** 1 Department of Agricultural Biotechnology, Faculty of Agriculture, Akdeniz University, Antalya 07058, Türkiye; nsourwaed1@gmail.com; 2 Graduate School of Natural and Applied Sciences, Akdeniz University, Antalya 07058, Türkiye; 3 Photosynthesis and Environment Team, Institute of Bioscience and Biotechnology of Aix-Marseille University, CEA/CNRS/AMU, 13108 Saint-Paul-lez-Durance, France; xenie.johnson@cea.fr

**Keywords:** *Dysmorphococcus*, *Haematococcus*, salt stress, fresh water stress, carotenoids, local strains

## Abstract

Microalgae are some of the organisms that produce carotenoids, which are high value metabolites. However, algae produce carotenoids under stress conditions and there is a need for more practical and economical ways to increase carotenoid accumulation in algae. *Haematococcus lacustris* has been one of the most important species for the production of industrial-scale carotenoids in biotechnology. In recent years, new algal species have been shown to accumulate carotenoids as alternative resources of carotenoids; *Dysmorphococcus globosus* is one of them. In a previous study, we isolated and identified for the first time a *D. globosus* strain in Türkiye. In the current study, we tested different stress conditions on carotenoid accumulation on *D. globosus*-KINIK and a *Haematococcus* sp.-KINIK strain that was isolated along with it from the same water sample. We partially sequenced the 18S rRNA gene of the newly isolated *Haematoccoccus* strain; it is 96% identical to *Haematococcus lacustris*. Further studies are necessary to determine the species of our strain. We tested three stress conditions: (i) salt stress by adding 2 g L^−1^ NaCl, (ii) fresh water stress by replacing growth media with fresh water, and iii) sea water stress by replacing growth media with sea water. Salt stress and fresh water stress have been shown to increase carotenoids in *Haematococcus* strains in the literature; our *Haematococcus* strain also responded well to these stresses and accumulated carotenoids. To our knowledge, none of these stresses have been tested for *Dysmosphococcus* strains in the literature. Our results show that *D. globosus*-KINIK also accumulates carotenoids under salt stress and fresh water, with salt stress being more effective. We also tested different growth media on the growth of *D. globosus*-KINIK and showed that it can grow without vitamin supplementation. Our study reveals local strains of carotenogenic algae in Türkiye for consideration for industrial-scale carotenoid production.

## 1. Introduction

In recent years, microalgae have attracted increasing attention because of their potential applications in the food, pharmaceutical, cosmetic, nutraceutical, and renewable energy industries. Owing to their unique biochemical composition, microalgae are considered valuable biological resources in modern biotechnology, as they are rich in proteins, carbohydrates, lipids, vitamins, minerals, and various bioactive compounds, including carotenoids [1,2]. Their low requirements for arable land and freshwater, the possibility of year-round cultivation, and their lack of competition with conventional crops make microalgae a sustainable biomass source, particularly in regions with limited resources [3]. In addition, microalgae are among the major photosynthetic organisms on Earth and produce valuable pigments, such as lutein, astaxanthin, and β-carotene, as well as phycobiliproteins and chlorophylls [4].

Carotenoids are a class of lipid-soluble pigments responsible for the yellow, orange, and red coloration of many plants and microorganisms and represent one of the most widespread pigment groups in nature. According to their chemical structure, carotenoids are classified into two major groups: carotenes and xanthophylls [5,6]. These carotenoids exhibit strong antioxidant properties and contribute to the prevention of oxidative damage and chronic diseases, including cardiovascular disorders, age-related macular degeneration, and cancer. Since humans are unable to synthesize these compounds, they must be obtained through dietary sources [3,5,7].

Reactive oxygen species (ROS) are highly reactive molecules generated as natural byproducts of cellular metabolism, mainly in chloroplasts and mitochondria during photosynthesis and electron transport processes. At low concentrations, ROS act as important signaling molecules involved in various cellular processes, including lipid accumulation in microalgae [8]. However, environmental stress conditions can lead to excessive ROS production, resulting in oxidative damage to DNA, proteins, lipids, and photosynthetic pigments [9]. ROS occur in several forms, including superoxide anions (O_2_·^−^), hydrogen peroxide (H_2_O_2_), singlet oxygen (^1^O_2_), and hydroxyl radicals (HO·) [10]. To counteract oxidative stress, microalgae synthesize antioxidant compounds, particularly carotenoids, which play a crucial role in protecting cells from ROS-induced damage.

Astaxanthin (3,3′-dihydroxy-β,β′-carotene-4,4′-dione), a xanthophyll carotenoid containing hydroxyl and keto groups, exhibits superior antioxidant activity compared with many other carotenoids [11]. Owing to its strong antioxidant properties, natural astaxanthin has attracted considerable interest in the nutraceutical, cosmetic, healthcare, and aquaculture industries [12,13]. In addition to its beneficial effects on human health, astaxanthin supplementation has been reported to improve antioxidant capacity, immune responses, yolk pigmentation, meat quality, and resistance to environmental stress in poultry [14,15,16]. Astaxanthin is synthesized from β-carotene through the sequential action of several enzymes, including phytoene synthase (PSY), phytoene desaturase (PDS), lycopene cyclase (LCY), β-carotene hydroxylase (CHY), and β-carotene ketolase (BKT), which play essential roles in carotenoid biosynthesis in *Haematococcus lacustris* [17,18]. Phytoene synthase (PSY), which catalyzes the first committed and rate-limiting step of carotenoid biosynthesis, plays a central role in regulating carotenoid accumulation. The expression of the *PSY* gene is strongly influenced by environmental factors, particularly light, which modulates carotenoid production through complex signaling pathways. Light-mediated regulation of *PSY* expression has been shown to affect carotenoid accumulation and photosynthetic adaptation, highlighting the importance of this gene in the cellular response to changing environmental conditions [19]. β-Carotene hydroxylase (CHY), encoded by the *CHY* gene, plays a key role in astaxanthin biosynthesis by catalyzing the hydroxylation of β-carotene to zeaxanthin and the conversion of canthaxanthin to astaxanthin [18]. In *Haematococcus lacustris*, nutrient stress, high light intensity, salinity, and sodium acetate induced the expression of carotenoid biosynthesis genes, including *PSY* and *CHY*; however, the magnitude and timing of gene induction varied depending on the stress conditions [20].

*Haematococcus lacustris* is a unicellular freshwater green microalga belonging to the phylum Chlorophyta and is widely recognized as one of the most efficient natural producers of astaxanthin [21,22]. Its life cycle comprises vegetative green cells and red cysts rich in astaxanthin [17,23]. Under unfavorable environmental conditions, such as nutrient limitation, high light intensity, and salinity stress, vegetative cells lose their flagella and differentiate into non-motile cysts, in which secondary carotenoids, particularly astaxanthin, accumulate [24]. Owing to its remarkable carotenoid-producing capacity, *Haematococcus* spp. has become the most extensively studied model species for investigating astaxanthin biosynthesis in microalgae.

Various environmental and nutritional factors, including organic carbon supplementation [25], nitrogen and phosphorus deprivation [26,27], light intensity and temperature [28], salinity [29], and CO_2_ concentration [30], have been shown to significantly influence astaxanthin accumulation and carotenoid productivity in *Haematococcus* spp. In addition, the combined effects of environmental factors such as light intensity, temperature, and cell density have also been reported to promote astaxanthin accumulation [31]. These stress conditions alter photosynthetic activity and promote ROS production, thereby activating carotenogenesis pathways. Furthermore, signaling compounds such as melatonin and putrescine have been shown to enhance stress responses and support carotenoid biosynthesis through oxidative and hormonal regulation mechanisms [32].

In addition to *Haematococcus* spp., other green microalgae have recently attracted attention because of their ability to accumulate carotenoids under stress conditions. Among them, *Dysmorphococcus globosus* has emerged as a promising species with potential biotechnological applications, although it remains poorly characterized.

*Dysmorphococcus globosus* is a freshwater green microalga belonging to the phylum Chlorophyta, class Chlorophyceae, and family Phacotaceae [33]. Members of the genus *Dysmorphococcus* are characterized by the presence of two equal flagella emerging through separate apertures in the rigid lorica, a feature that distinguishes them from related taxa [34]. Cells are generally spherical and range from 10 to 50 μm in diameter, although most measure 20–25 μm, and typically divide into 4–8 daughter cells, with divisions into as many as 16 cells occasionally reported [34,35]. The cells contain a green cup- or urn-shaped chloroplast occupying a large proportion of the cellular volume, together with one or more pyrenoids and a small eyespot [34,36]. Although motile stages have been described, they are rarely observed and are characterized by the absence of a thick extracellular matrix [35]. Under unfavorable environmental conditions, *D. globosus* can produce motile zoospores and non-motile aplanospores [36].

Despite the wide distribution of the genus, studies on *Dysmorphococcus* remain limited and have mainly focused on taxonomy, phylogeny, morphology, and physiology. Nevertheless, recent studies have highlighted its potential as a natural source of carotenoids and astaxanthin. Under zinc stress, *D. globosus* cells undergo a transition from green to orange-red because of carotenoid accumulation, with the highest astaxanthin content (0.311 mg g^−1^) being obtained at 45.5 mg L^−1^ Zn^2+^, accompanied by increased canthaxanthin and zeaxanthin levels [37]. Similarly, the Himalayan strain *D. globosus*-HI has been reported to exhibit high carotenoid productivity, reaching an astaxanthin concentration of 391 mg L^−1^ and a productivity of 15.6 mg L^−1^ day^−1^ [38]. In addition, carotenoid-rich biomass of *Dysmorphococcus* was shown to contain approximately 1.2 mg g^−1^ dry weight canthaxanthin and 0.7 mg g^−1^ dry weight astaxanthin, predominantly in the form of astaxanthin monoesters [39]. Despite its promising biotechnological potential, *D. globosus* remains poorly studied at the molecular level. Recently, the species was reported for the first time in Türkiye based on morphological and molecular analyses [40].

The strains analyzed in this study, belonging to genus *Haematococcus* and *Dysmorphococcus*, were newly isolated from Antalya region and named *Haematococcus* sp.-KINIK (this study) and *D. globosus*-KINIK [40]. Previous studies have demonstrated that salinity stress enhances astaxanthin accumulation in *Haematococcus* sp. [29], whereas its effects on *D. globosus* remain unknown. Main objective of this study was to test if carotenoid accumulation in liquid cultures of *D. globosus*-KINIK can be increased by adding NaCl to the cultures or transferring the cells to sea water. To our knowledge, this is the first study to test these stress conditions on *D. globosus*. Therefore, in the present study, *D. globosus*-KINIK was exposed to different stress treatments to evaluate its growth and carotenoid accumulation potential. *Haematococcus* sp.-KINIK and *Chlamydomonas reinhardtii* were included as positive and negative controls, respectively. In addition, the expression of the carotenoid biosynthesis genes phytoene synthase (*PSY*) and carotene hydroxylase (*CHY*) was analyzed in *Haematococcus* sp.-KINIK to investigate their involvement in stress-induced carotenoid accumulation.

## 2. Results

Before beginning the stress applications, we determined the optimal growth media and maximum cell numbers we can obtain for the newly isolated strains *Dysmorphococcus globosus*-KINIK and *Haematococcus* sp.-KINIK. We cultivated cells in known algal media such as TAP, BG11 or BBM, firstly without vitamin supplementation. If we did not get any growth or less than 1 × 10^6^ cells/mL in liquid cultures, then we added vitamins to the media. These experiments also allowed us to obtain growth curves for each strain. These growth curves were necessary for the timing of the application of stress conditions discussed below.

### 2.1. Determination of Optimal Growth Media

**Figure 1:**
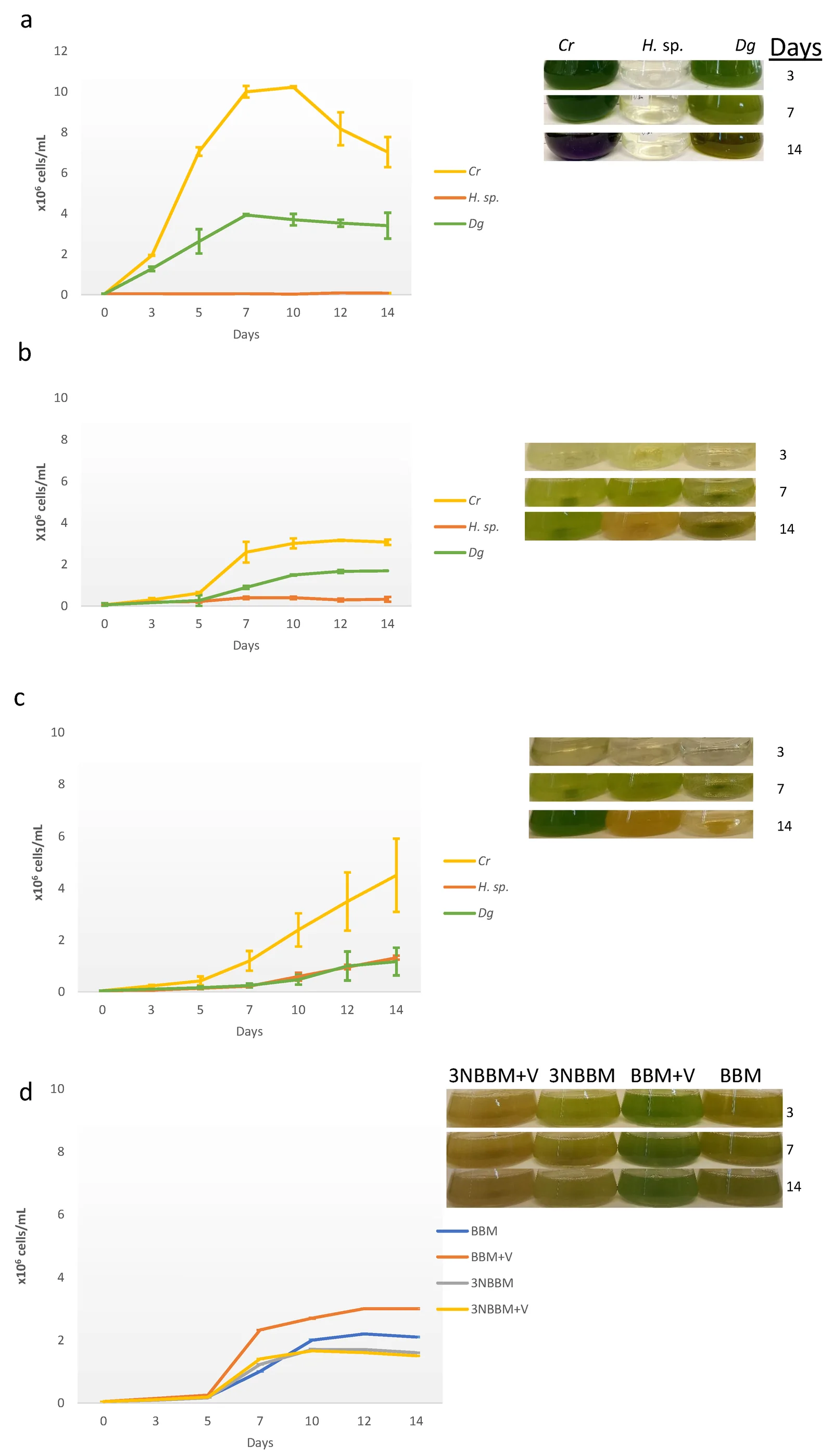
Growth of the strains in different media; (**a**) TAP, (**b**) BG11 and (**c**) BBM. Growth experiments were repeated at least three times. Results show the average of three experiments; error bars show standard deviation. (**d**) Growth of *Haematococcus* sp.-KINIK in different versions of BBM. Insets show the culture flasks of one of the growth experiments. *Cr*, *Chlamydomonas reinhardtii; H.* sp, *Haematocoocus* sp.-KINIK, *Dg*, *Dysmorphococcus globosus*-KINIK.

We cultured the strains in TAP (Figure 1a), BG11 (Figure 1b) and BBM (Figure 1c) media. *Chlamydomonas reinhardtii* was used as a reference; known to grow optimally in TAP media [41]. Growth experiments showed that *D. globosus*-KINIK also grows best in TAP media (Figure 1a), comparing to BG11 (Figure 1b) and BBM (Figure 1c). *D. globosus*-KINIK reached a cell density of almost 4 × 10^6^ cells/mL at the seventh day of cultivation; growth in the other media was much lower and slower. *Haematococcus* sp.-KINIK strain did not grow well in TAP or BG11 media (Figure 1b and Figure 1c, respectively). However, it reached a cell density of almost 1 × 10^6^ cells/mL after fourteen days in BBM (Figure 1c). To increase growth of the *Haematococcus* sp.-KINIK strain, we added vitamins to BBM media. The addition of vitamins resulted in better growth; cell density reached almost 3 × 10^6^ cells/mL at the twelfth day of cultivation (Figure 1d). The *Haematococcus* sp.-KINIK strain did not grow well in 3NBBM with or without vitamins (Figure 1d). From these growth experiments, we concluded that *D. globous*-KINIK can be grown without vitamin supplementation, which will be beneficial in larger scale cultivations. However, the *Haematococcus* sp.-KINIK strain could not be grown without vitamin supplementation.

After determining the most suitable media for each strain, we cultivated *D. globosus*-KINIK in TAP and *Haematococcus* sp.-KINIK in vitamin-supplemented BBM (BBM+V) in further experiments.

### 2.2. Stress Applications

We wanted to test if we could increase carotenoid accumulation using simple and more economical methods. Most of the methods seen in the literature include high light treatment along with other stress factors such as nutrient removal or salt application. We tried different stresses without using high light, as discussed below. Culture color transition from green to red was monitored as a phenotypic proxy for astaxanthin production and accumulation.

#### 2.2.1. Salt Application at Mid Log Phase

**Figure 2:**
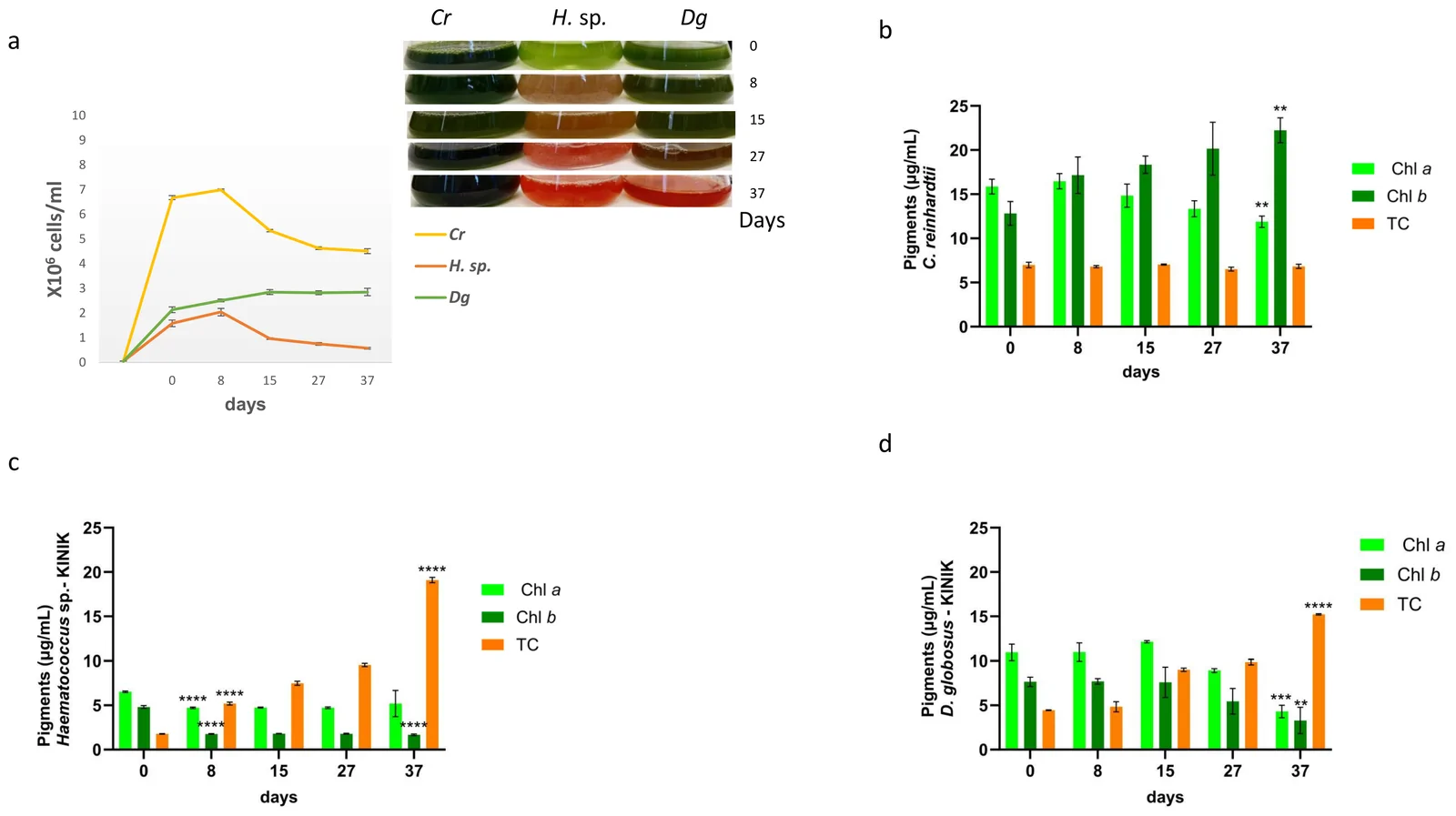
Salt application to mid-log-phase cultures. Day 0 is the day stress was applied. (**a**) Cell number and condition of cultures. Pigment amounts in (**b**) *C. reinhardtii*, (**c**) *Haematococcus* sp.-KINIK, and (**d**) *D. globosus*-KINIK. Technical triplicates from 3 biological replicates were averaged and used for the statistical analysis. Unpaired *t* tests were applied to find statistical significance at 2 time points, comparing day 8 and day 37 to day 0. Statistical significance is represented by the number of asterisks: ** *p* < 0.01 (moderately high significant difference), *** *p* < 0.001 (highly significant difference), and **** *p* < 0.0001 (extremely high significant difference). When no asterisk(s) are shown, there was no statistical significance found. Chl *a,* Chlorophyll *a*; Chl *b,* Chlorophyll *b*; TC, total carotenoid.

*D. globosus*-KINIK and *C. reinhardtii* cells were grown in TAP for five days and *Haematococcus* sp.-KINIK cells were grown in BBM+V for nine days before salt application. All of the cultures were green on these days, indicated as day “0” (Figure 2a, inset). Salt application did not increase carotenoids in *C. reinhardtii*. However, there was an increase in chlorophyll *b* in this alga (Figure 2b). Carotenoid amount increased in *Haematococcus* sp.-KINIK after eight days and reached a maximum amount at day thirty-seven. The maximum carotenoid amount was 20 µg/mL. After thirty-seven days there was no increase. The chlorophyll *b* amount decreased under salt stress in *Haematococcus* sp.-KINIK (Figure 2c). Salt application increased carotenoids in *D. globosus*-KINIK, also reaching 15 µg/mL on day thirty-seven (Figure 2d), which is lower than *Haematococcus* sp.-KINIK (Figure 2c).

#### 2.2.2. Salt Application at Stationary Phase

**Figure 3:**
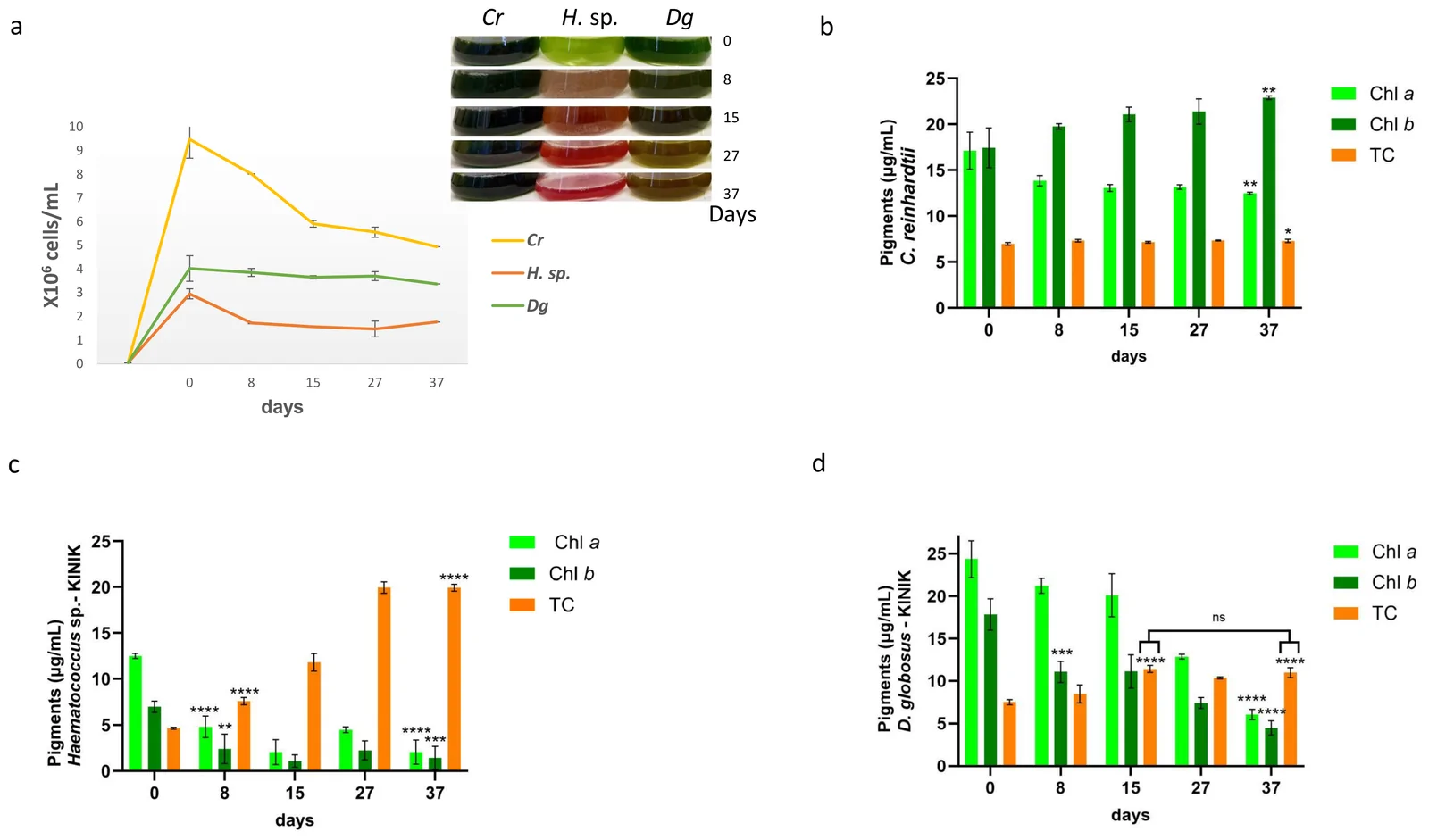
Salt application to stationary phase cultures. Day 0 is the day stress was applied. (**a**) Cell number and condition of cultures. Pigment amounts in (**b**) *C. reinhardtii*, (**c**) *Haematococcus* sp.-KINIK, and (**d**) *D. globosus*-KINIK. Technical triplicates from 3 biological replicates were averaged and used for the statistical analysis. Unpaired *t* tests were applied to find statistical significance at 2 time points, comparing day 8 and day 37 to day 0. Statistical significance is represented by the number of asterisks: * *p* < 0.05 (significantly different), ** *p* < 0.01 (moderately high significant difference), *** *p* < 0.001 (highly significant difference), and **** *p* < 0.0001 (extremely high significant difference). When no asterisk(s) are shown, there was no statistical significance found. Chl *a,* Chlorophyll *a*; Chl *b,* Chlorophyll *b*; TC, total carotenoid.

The same salt treatments were applied to ten-day-old *D. globosus*-KINIK and *C. reinhardtii* and twelve-day-old *Haematococcus* sp.-KINIK cultures. The cells were green (Figure 3a, inset); there were more cells in the cultures before the salt application compared to mid-log cultures (compare cell numbers in Figure 2a and Figure 3a), indicated as day “0” (Figure 3a). There was no increase in carotenoid accumulation in *C. reinhardtii*; again the chlorophyll *b* amount increased in this alga (Figure 3b). Interestingly, *Haematococcus* sp.-KINIK accumulated carotenoids much earlier, at day fifteen, and the maximum was reached at day twenty-seven (Figure 3c). Comparing to mid-log-phase salt application, the maximum amount was reached ten days earlier (compare Figure 2c and Figure 3c); comparatively, for the mid-log-phase salt application, even the color of the flask was not red yet at day fifteen (Figure 2a, inset). *D. globosus*-KINIK also accumulated more carotenoids; however, this was less compared to the mid-log-phase salt application (compare Figure 2d and Figure 3d), with the maximum quantity already reached by day 15 (no significant difference in carotenoid accumulation between day 15 and day 37).

#### 2.2.3. Fresh Water Application

**Figure 4:**
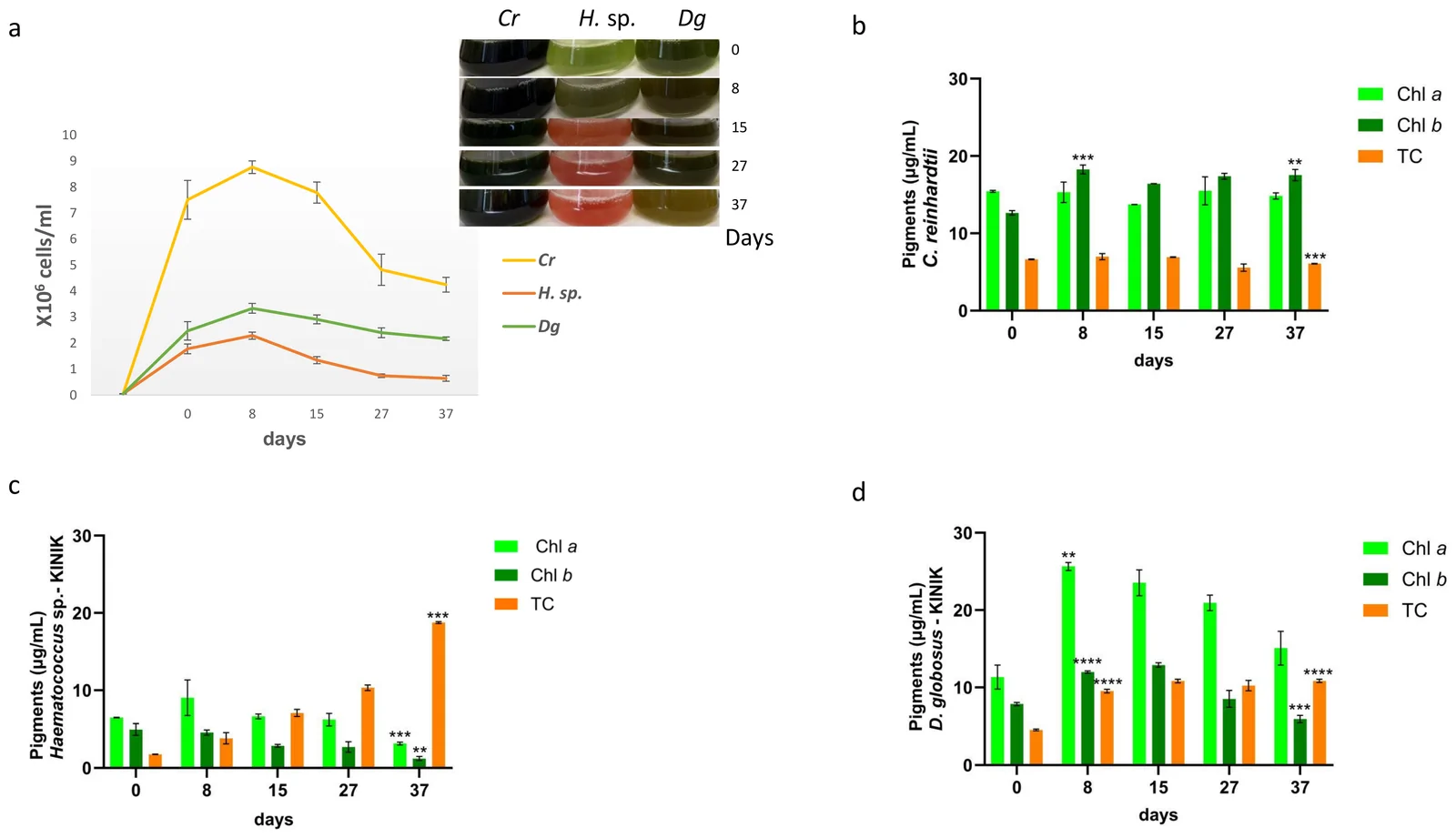
Fresh water application to mid-log-phase cultures. Day 0 is the day stress was applied. (**a**) Cell number and condition of cultures. Pigment amounts in (**b**) *C. reinhardtii*, (**c**) *Haematococcus* sp.-KINIK, and (**d**) *D. globosus*-KINIK. Technical triplicates from 3 biological replicates were averaged and used for the statistical analysis. Unpaired *t* tests were applied to find statistical significance at 2 time points, comparing day 8 and day 37 to day 0. Statistical significance is represented by the number of asterisks: ** *p* < 0.01 (moderately high significant difference), *** *p* < 0.001 (highly significant difference), and **** *p* < 0.0001 (extremely high significant difference). When no asterisk(s) are shown, there was no statistical significance found. Chl *a,* Chlorophyll *a*; Chl *b,* Chlorophyll *b*; TC, total carotenoid.

Fresh water was applied only to mid-log-phase cultures, since the maximum amount of carotenoids was obtained in mid-log cultures of *D. globosus*-KINIK with salt application. The response of *Haematococcus* sp.-KINIK and maximum amount of carotenoids accumulated were similar to salt application results. However, maximum carotenoid accumulation was on day thirty-seven; later than the day twenty-seven result observed for the stationary phase salt application (compare Figure 3c and Figure 4c). Fresh water increased carotenoids in *D. globosus*-KINIK also, however the maximum amount was only around 10 µg/mL. The reference strain *C. reinhardtii* again did not increase carotenoid accumulation (Figure 4b).

#### 2.2.4. Sea Water Application

**Figure 5:**
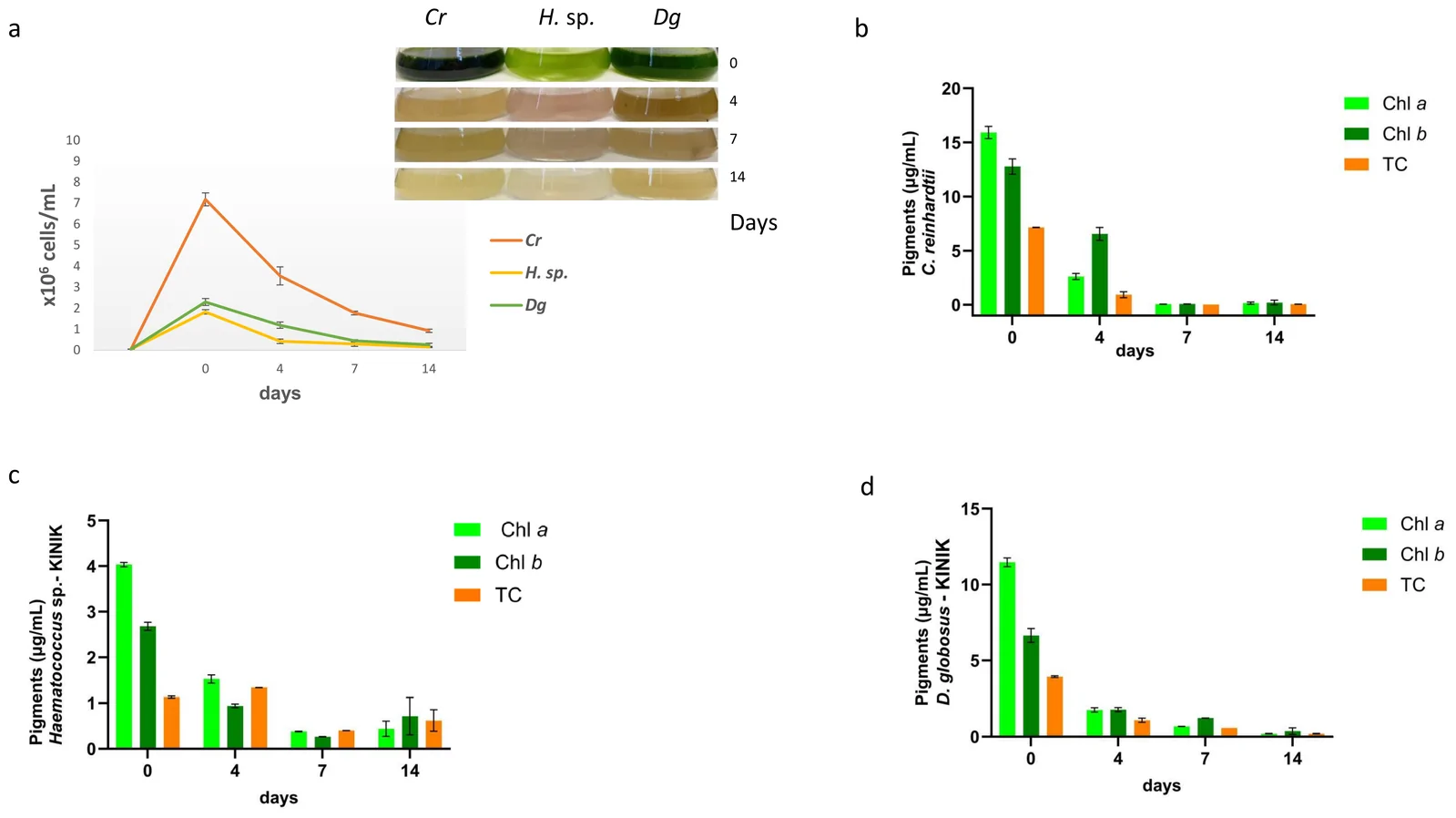
Sea water application to mid-log-phase cultures. Day 0 is the day stress was applied. (**a**) Cell number and condition of cultures. Pigment amounts in (**b**) *C. reinhardtii*, (**c**) *Haematococcus* sp.-KINIK, and (**d**) *D. globosus*-KINIK. Chl *a,* Chlorophyll *a*; Chl *b,* Chlorophyll *b*; TC, total carotenoid.

Cells were grown to mid-log phase before application of sea water stress. The cells were green (Figure 5a, inset day “0”). However, the cells died rapidly after sea water application (Figure 5a). All the cultures bleached and all the pigments diminished quickly (Figure 5). We concluded that sea water was not suitable for increasing carotenoids for any of the strains.

**Figure 6:**
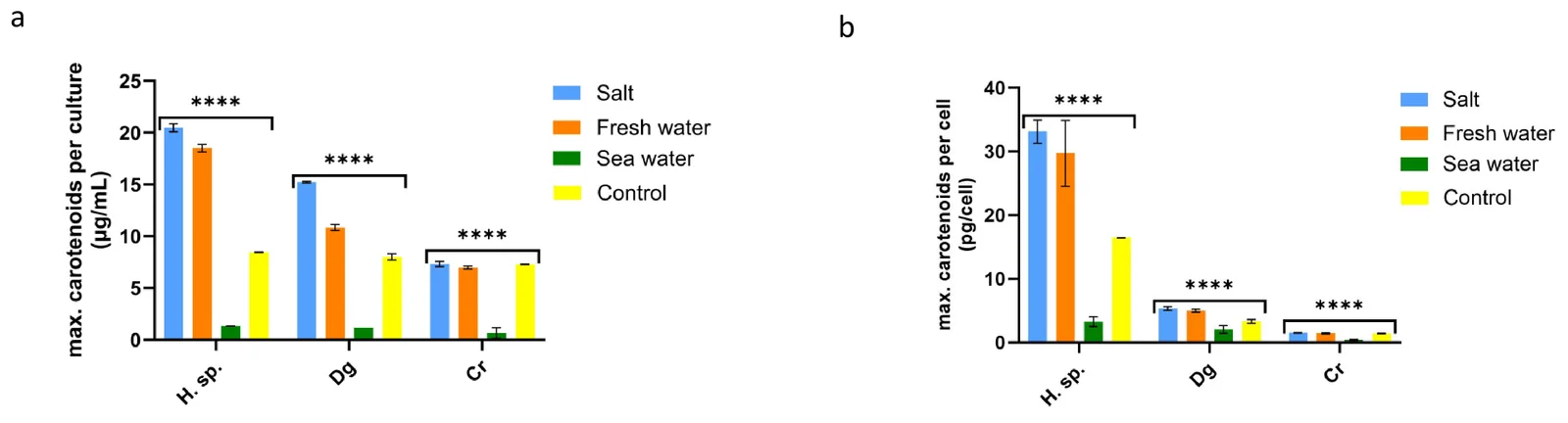
Comparison of maximum amount of carotenoids under different stress conditions and control (no treatment). (**a**) Amount of carotenoids at day thirty-seven per mL of culture. (**b**) Amount of carotenoids at day thirty-seven per cell. A 2-way ANOVA using a mixed model was generated to test effects between treatments, strains and treatment/strain interactions. Statistical significance is represented by the number of asterisks: extremely highly significant (**** *p* < 0.0001).

In summary, the best condition for increasing carotenoids for *D. globosus*-KINIK was salt application to mid-log-phase cultures. The maximum amount of carotenoids was 15 µg/mL for this application. This amount was obtained after thirty-seven days in salt stress. Furthermore, the percentage effects were found to be extremely highly significant, showing real differences in pigment accumulation in response to each stress and between species. Cultures without any stress application, just keeping them in the media for thirty-seven days, had increased amount of carotenoids, but the amounts were much lower than salt and fresh water applications (compare Figure 6 control values with values on day “0” in Figure 2, Figure 3 and Figure 4). Although salt stress increased carotenoids in *D. globosus*-KINIK, the *Haematococcus* sp.-KINIK strain had more carotenoids on a per mL and per cell basis (Figure 6a,b).

### 2.3. Molecular Identification and Cell Morphology

**Figure 7:**
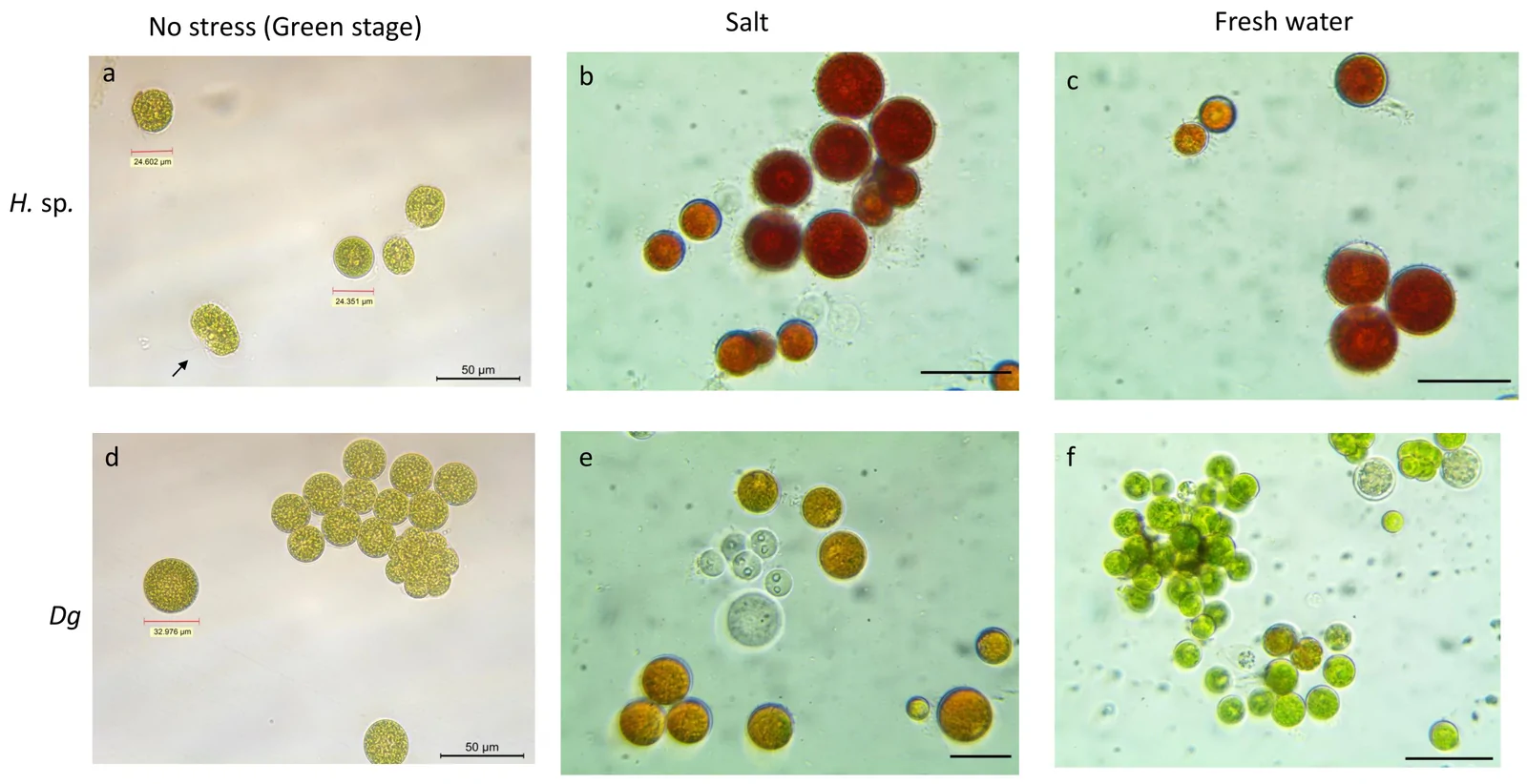
Microscope images of cells in green and red stages. Images of cells were taken on day seven in cultures with no stress (green stage) and thirty-seven days after stress applications. In green stage images, *Haematococcus* sp.-KINIK was grown in BBM+V and *D. globosus*-KINIK was grown in TAP media. *H.* sp., *Haematococcus* sp.-KINIK; Dg, *D. globosus*-KINIK. All scale bars are 50 µm.

We described *D. globosus*-KINIK strain previously [40]. In the current study, *Haematococcus* sp.-KINIK 18S rRNA gene was amplified by PCR and the 958 bp region was sequenced. This sequence was used in NCBI Blast analysis. Top three hits of the Blast results are shown in Table 1. Our strain is 96.14% identical to three *H. lacustris* strains; this value is not enough to identify our strain at the species level. However, Blast results along with cell morphology (Figure 7) clearly suggest this strain belongs to the genus *Haematococcus*.

Cells were examined by light microscope before and after stress treatments. *Haematococcus* sp.-KINIK cells were around 30 µm in diameter and oval shaped. The cells had two flagella at the green stage and were motile (Figure 7a, shown with arrow). The cells turned red under stress applications (Figure 7b,c). The *D. globosus*-KINIK cells were also around 30 µm and round shaped. We did not observe any flagella in these cells. The cells turned to a reddish orange color after stress treatments (Figure 7e,f), concurrent with less amounts of carotenoids compared to the *Haematococcus* sp.-KINIK strain, which has a unique red color (Figure 7b,c).

### 2.4. PSY and CHY Transcripts Under Stress Conditions

**Figure 8:**
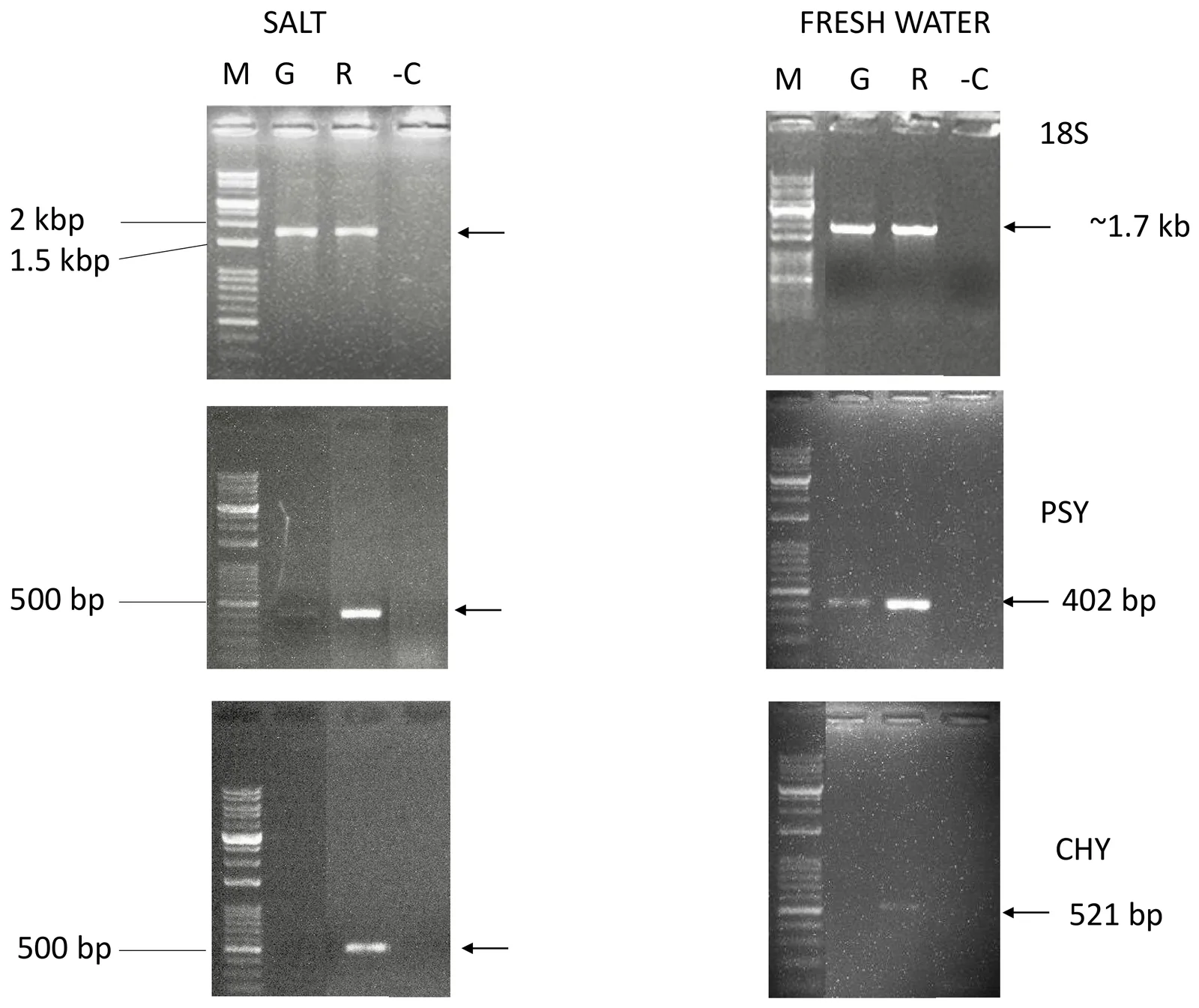
Agarose gel of RT-PCR products of *PSY* and *CHY* in green (G) and red (R) stages. 18S rRNA was used as control. -C, negative control (no cDNA template was added).

We used RT-PCR to determine the transcript accumulation of two of the genes in the carotenoid biosynthesis pathway in *Haematococcus* sp.-KINIK before and after stress treatments. We used 18S rRNA as the control; the amount of this RNA was the same in the green stage and red stage under both salt and fresh water applications, as expected (Figure 8, top). Amplification of the *PSY* transcript was more abundant under both stress conditions than under no stress conditions (Figure 8, middle panels, 402 bp product). The *CHY* transcript was not detected in no stress conditions. *CHY* increased under salt stress, however there was a slight increase under fresh water stress compared to salt stress (Figure 8, bottom, 521 bp product). Since the maximum carotenoid amount was also less under fresh water treatment compared to salt treatment (Figure 6a), it is plausible to suggest the lower *CHY* transcript amount is in correlation with lower carotenoid amounts. However, the technique we have used is most accurate to show that *PSY* and *CHY* are induced under stress to produce carotenoids.

## 3. Discussion

There is an increasing demand for microalgae and value-added compounds obtained from microalgae, such as carotenoids, lipids, etc., in food, feed and cosmetics industries alike [15]. Algae also contain high quality protein, which makes them desirable [42]. Carotenoids, specially astaxanthin, are natural antioxidants that protect cells from oxidative stress and have potential for prevention of age-related diseases [43]. Although the market share of synthetic astaxanthin is higher than natural astaxanthin, natural astaxanthin has been reported to be stronger and safer than synthetic astaxanthin; additionally, consumers demand natural astaxanthin [44]. Microalgae can provide these natural compounds, however there is a need for more practical and economical ways for increasing carotenoids in algae.

In the search for carotenogenic microalgal species, we isolated a *Haematococcus* and a *Dysmorphococcus* strain, *Haematococcus* sp.-KINIK and *D. globosus*-KINIK, respectively, from Antalya region. Molecular identification of *D. globosus*-KINIK has been done recently [40]. In this study, partial sequencing of the 18S rRNA gene of *Haematococcus* sp.-KINIK has been done. The sequence has 96.14% identity to *H. lacustris* strains (Table 1). Further studies, such as sequencing the full-length 18S rRNA gene and ITS regions, are necessary to conclude the species of our strain. However, the cell morphology (Figure 7) and 18S sequencing suggest it belongs to the genus *Haematococcus*. We expect these local strains could be well adapted to the climatic conditions of Antalya and could be used in large-scale outdoor cultivation. Sourcing and cultivation of local strains of microalgae can provide to the economy of local communities in business endeavors.

*Dysmorphococcus globosus* has been newly identified as a high-astaxanthin-containing microalga [38]. There are works in the literature for increasing carotenoids in *Hematococcus lacustris* [29,45]; however no reports have been found for *D. globosus*. To increase carotenoids using a practical method, we applied different stress conditions to our newly isolated strains. To the best of our knowledge, this is the first study to test the effects of salt application on carotenoid accumulation in a *Dysmorphococcus* strain. Our study comprehensively demonstrated the effects of different osmotic stress treatments on carotenoid production in the locally isolated strains *Haematococcus* sp.-KINIK and *Dysmorphococcus globosus*-KINIK. The *Haematococcus* sp.-KINIK strain had more carotenoids per mL of culture and more carotenoids per cell compared to *D. globosus*-KINIK (Figure 6a and Figure 6b, respectively). However, we were able to cultivate *D. globosus*-KINIK without vitamin supplementation, which is more advantageous for large-scale applications. The findings indicate that, in particular, salt treatment represents a biologically effective, economically feasible, and promising approach for industrial-scale production by enhancing carotenoid accumulation. The ability of *D. globosus*-KINIK to grow successfully in culture media without the need for vitamin supplementation, together with its faster growth compared with *Haematococcus* sp.-KINIK, demonstrates its potential to be considered as an alternative microalgal source for carotenoid production.

The reverse transcription PCR analyses performed in this study revealed that salt stress simultaneously activated the expression of the *PSY* and *CHY* genes in *Haematococcus* sp.-KINIK, whereas distilled water stress activated only the *PSY* gene and did not markedly increase *CHY* expression (Figure 8). Notably, the total carotenoid contents under both stress conditions were at similar levels, suggesting that this difference in gene expression may also result in differences in pigment composition. This finding is important because it indicates that the types of osmotic stress may regulate the carotenoid biosynthetic pathway differently, not only in terms of the quantity of carotenoids produced but also regarding which carotenoid species are synthesized. We plan to examine the carotenoid species by HPLC analyses in future studies.

Our study was done without highlight application and CO_2_ supplementation, making the growth conditions more practical and economical; we suggest using these conditions might have further enhanced cell density in the initial growth phase (green stage before stress applications). However, since *D. globosus* cells are larger, we concluded a cell density of 4×10^6^ cells/ml is sufficient for our studies. High light and CO_2_ supplementation conditions could be tested in the future if they will further increase carotenoid amounts. Additionally, photosynthetic activities under stress conditions could be studied in future. Our chlorophyll measurements show that there was some decrease in both chlorophyll *a* and *b* amounts, however the chlorophyll *a*/*b* ratio did not change drastically in both *Haematococcus* sp.-KINIK (Figure 2c and Figure 3c) and *Dysmorphococcus globosus*-KINIK (Figure 2d and Figure 3d) under salt stress. This suggests photosynthetic activity may have been reduced but not affected drastically in these algae. This hypothesis will be tested in future studies. However, the chlorophyll *a*/*b* ratio was changed in *C. reinhardtii*, suggesting photosynthetic activity might be affected drastically in this alga (Figure 2b and Figure 3b). However, fresh water treatment did not change the chlorophyll *a*/*b* ratio in *C. reinhardtii* (Figure 4b).

## 4. Materials and Methods

### 4.1. Microalgal Strains and Molecular Identification

*Haematococcus* sp.-KINIK and *Dysmorphococcus globosus*-KINIK used in this study were isolated from the same natural water sample collected in February 2023 from a small rainwater rock pool in Kaş, Antalya, Türkiye (36°21′24.28″ N, 29°19′13.41″ E; ~60 m above sea level). The water exhibited a distinct red coloration at the time of sampling. After collection, the samples were transported to the laboratory, where monoclonal strains were isolated and cultured in vitro. *D. globosus*-KINIK was identified based on 18S rRNA sequencing and morphological analyses, as previously described [40], whereas *Haematococcus* sp.-KINIK was identified based on 18S rRNA sequencing and morphological analysis in this study. Briefly, primers 16S1N (5′-TCCTGCCAGTAGTCATATGC-3′) and 16S2N (5′-TGATCCTTCT/CGCAGGTTCAC-3′) was used to amplify the 18S rRNA gene [46]. A single band of ~1700 bp was obtained and sequenced by Sanger sequencing (BM Labosis, Ankara, Turkey). The 758 bp read that was obtained was used to identify the *Haematococcus* strain.

### 4.2. Culture Media and Growth Conditions

Different culture media were prepared to determine the optimal growth conditions for the isolated strains. Vitamin-supplemented Bold’s Basal Medium (BBM + V), a general freshwater medium, was prepared according to a modified protocol based on Bischoff and Bold (1963) and Starr and Zeikus (1993) [47,48]. To prepare the vitamin solution, thiamine (0.1 g), biotin (25 × 10^−6^ g) and vitamin B12 (15 × 10^−6^ g) were dissolved in 100 mL distilled water and filter sterilized. A total of 1 mL of this vitamin solution was added to 1 L autoclaved and cooled media. BG-11 medium was prepared following the method described by Rippka and Herdman (1992) [49], whereas Tris–Acetate–Phosphate (TAP) medium was prepared according to Gorman and Levine (1965) [41]. Cultures were maintained under controlled conditions at 25 ± 1 °C in 100 mL Erlenmeyer flasks under white fluorescent LED illumination (~60 μmol photons m^−2^ s^−1^), continuous light, and agitation at 120 rpm. There was no extra air supplementation to the cultures. The initial cell concentration was adjusted to 5 × 10^4^ cells mL^−1^, and cultures were maintained for 14 days. Cell density was determined by using a hemocytometer under a light microscope, and growth curves were generated from samples collected on days 0, 3, 5, 7, 10, 12, and 14.

To determine the most suitable culture medium for each species, *Haematococcus* sp.-KINIK and *D. globosus*-KINIK were cultivated in BBM + V, BG-11, and TAP media under standard conditions. In addition, comparative growth analyses of *Haematococcus* sp.-KINIK were performed in 3N-BBM, 3N-BBM + V, and BBM + V media. The medium providing the highest cell growth was selected for subsequent experiments. All experiments were conducted in triplicate.

### 4.3. Gene Expression Analysis of Phytoene Synthase (PSY) and Carotene Hydroxylase (CHY)

Total RNA was isolated from *Haematococcus* sp.-KINIK cultures collected before (green phase) and after (red phase) stress treatment using the RNeasy Plant Mini Kit (Cat. No. 74904, QIAGEN, Hilden, Germany) according to the manufacturer’s instructions. RNA samples were diluted 1:10 with RNase-free water, and their integrity was assessed by 1% agarose gel electrophoresis in 1× TAE buffer stained with ethidium bromide (EtBr).

First-strand cDNA was synthesized from 1 μg of total RNA using the OneScript^®^ Plus Reverse Transcriptase kit (Cat. No. E018, ABM, Richmond, BC, Canada). The synthesized cDNA was used as a template for PCR amplification (total of 35 cycles). Gene expression analysis was performed using primers specific for phytoene synthase (*PSY*; GenBank accession no. AY835634) (forward: 5′-ATGTACCATCCCAAGGCAAG-3′; reverse: 5′-CTGGACCAGGCCTACGAC-3′) and carotene hydroxylase (*CHY*; GenBank accession no. AF162276) (forward: 5′-CTACACCACAGCGGCAAGTA-3′; reverse: 5′-GCCTCACCTGATCCTACCAA-3′) [20], with 18S rRNA as the reference gene (18s-1N: 5′-TCCTGCCAGTAGTCATATGC-3′; 18s-2N: 5′-TGATCCTTCTCGCAGGTTCAC-3′) [46]. PCR products were run on agarose gel and visualized as in above conditions. A 1 kb Plus Opti-DNA ladder (Cat. No. G106, ABM, Canada) was used as the molecular size marker.

### 4.4. Pigment Analysis

Chlorophyll *a*, chlorophyll *b*, and total carotenoid contents were determined in *Haematococcus* sp.-KINIK, *D. globosus*-KINIK, and the control strain *C. reinhardtii* before (green phase) and after (red phase) stress treatment. Cell density was simultaneously determined using a hemocytometer (Hausser Scientific, Horsham, PA, USA). For pigment analysis, 2 mL culture samples were centrifuged at 10,000 rpm for 4 min, and the resulting cell pellets were resuspended in 1 mL of 100% methanol. Samples were disrupted using metal beads and incubated in the dark at 70 °C until complete pigment extraction. Following centrifugation at 14,000 rpm for 10 min, absorbance values were measured at 480, 652, 665, and 750 nm using a spectrophotometer. When necessary, samples were diluted with 100% methanol (1:5, *v*/*v*) prior to measurement. Chlorophyll *a*, chlorophyll *b*, and total carotenoid concentrations were calculated according to the equations described by Ritchie and Strickland [50,51,52].

Technical triplicates from 3 biological replicates were averaged and used for the statistical analysis. GraphPad Prism Version 10.4.2 was used for multiple *t*-test analysis—the tests were unpaired and assumed different SDs—using the Welch *t*-test. Pigment measurements from the time course were treated to unpaired *t* tests and always compared to day 0, with a focus on day 8 and day 37. Statistical significance is represented by the number of asterisks: * *p* < 0.05 (significantly different), ** *p* < 0.01 (moderately high significant difference), *** *p* < 0.001 (highly significant difference), and **** *p* < 0.0001 (extremely high significant difference).

For Figure 6, 2-way ANOVA using a mixed model was generated to test effects between treatments, strains and treatment/strain interactions. In all cases the percentage effects were found to be extremely highly significant (**** *p* < 0.0001).

### 4.5. Morphological Analysis

Morphological changes in *Haematococcus* sp.-KINIK and *D. globosus*-KINIK cultures were monitored by microscopic imaging at regular intervals. Samples collected during the green (active growth) and red (stress) phases were examined using Leica MC 190 HD and Leica DM750 light microscopes. Images were acquired using 20× and 40× objective lenses to evaluate stress-induced changes in cell morphology.

### 4.6. Stress Treatments

Three different stress treatments were applied to *Haematococcus* sp.-KINIK, *D. globosus*-KINIK, and the control strain *C. reinhardtii* to evaluate their effects on growth and carotenoid production. Cultures were also kept untreated and pigment measurements were done at the stationary phase (thirty-seven days after cultures were started) to compare with the stress treatments explained below.

#### 4.6.1. Salt Stress

Salt stress was induced by adding NaCl to the culture medium at a final concentration of 2 g L^−1^. The treatment was applied at two different growth stages to assess the effects of stress timing on cell growth and pigment accumulation. For *D. globosus*-KINIK and *C. reinhardtii*, salt stress was applied to five-day-old (mid logarithmic phase) and ten-day-old cultures (stationary phase), whereas for *Haematococcus* sp.-KINIK, the treatment was applied to nine-day (mid logarithmic phase) and twelve-day-old cultures (stationary phase).

#### 4.6.2. Fresh Water Stress

Cells were harvested by centrifugation, and the culture medium was replaced with 100 mL of autoclaved distilled water to induce osmotic stress. For *D. globosus*-KINIK and *C. reinhardtii*, five-day-old cultures were used, whereas for *Haematococcus* sp.-KINIK nine-day-old cultures were used.

#### 4.6.3. Sea Water Stress

Natural sea water collected from Konyaaltı Beach (Antalya, Türkiye) was filtered, autoclaved, and used to replace the culture medium after centrifugation of the cells, thereby exposing the cultures to osmotic stress. For *D. globosus*-KINIK and *C. reinhardtii*, five-day-old cultures were used, whereas for *Haematococcus* sp.-KINIK nine-day-old cultures were used.

## Figures and Tables

**Table 1:** Blast results of 18S rRNA Sequence of *Haematococcus* sp.-KINIK.

	Scientific Name	Max Score	Total Score	Query Cover	E Value	Percent Identity	Accession Length	Accession No. (Genebank)
*H.* sp.-KINIK 18S	*Haematococcus lacustris* strain NRMC-F	1543	1543	99%	0	96.14%	1936	PZ503066.1
	*Haematococcus lacustris* isolate Sogang	1543	1543	99%	0	96.14%	1790	MW730617.1
	*Haematococcus lacustris* isolate JNU35	1543	1543	99%	0	96.14%	1689	MH681993.1

## Data Availability

The data presented in this study are available in the article.
